# New Appliances and Things Medical

**Published:** 1901-05-25

**Authors:** 


### NEW APPLIANCES AND THINGS MEDICAL.

[We shall be glad to receive, at our Office, 28 & 29 Southampton Street, Strand, London, W.C., from the manufacturers, specimens of all new preparations and appliances which may be brought out from time to time.]

### THE CAMBRIDGE LEMONADE.

(Chivers & Sons, Limited, Histon, Cambridge)

This lemonade is stated to be prepared from selected Sicilian lemons, and is guaranteed to contain the natural refreshing constituents of the fresh ripe fruit, free from any added acid or other injurious ingredient. Our examination of the crystalline material of which it is composed leads us to confirm these statements, for it contains the ordinary organic salts and acids contained in lemon juice, and the flavour is that of the essential oils of lemon; a large proportion of the material consists of sugar. By dissolving a sufficiency of the lemon crystals in water a most delicious lemonade can be made at a moment's notice. Each packet contains sufficient to prepare two gallons of superior lemonade. A beverage thus prepared, and at an exceedingly moderate cost, ought to be a great boon to teetotallers.

### RONUK POLISHES.

("Ronuk," Limited, 83 Upper Thames Street, London, E.C.)

We have examined the following polishes, prepared by the above firm:—No. 1, household; No. 2, special brown boot; No. 3, special black; No. 4, harness composition, and finally the "special white." Each for the particular purpose for which it is intended is a first-rate preparation, cleanly, and imparting a high polish. Each is agreeably perfumed and economical in use, both as regards time and trouble. The "special white" should be particularly popular for cleaning and preserving all kinds of enamelled and glacé leather boots and shoes.

### FLAT CAMEL-HAIR SPONGE.

(John Milne, Galen Manufacturing Company, Limited, Ladywell, London, S.E.)

This new artificial sponge is of ingenious conception; it is composed of layers of matted camel's hair (camel-hair blanketing) contained in an outer casing of butter cloth, which is stitched through, or, as it were, quilted, so as to prevent lumping of the hair. It is claimed for this sponge that it offers the following advantages: (1) easy sterilisation by boiling; (2) great power of absorption; (3) elasticity and smooth surface; (4) cheapness. It is probable that if this substitute for the marine sponge has a future before it, it will be in important abdominal operations, for in such cases the uniformity of the surface will be less likely to do damage to the tender peritoneal epithelium than the very finest of Turkish sponges. Boiling would doubtless sterilise such a sponge, but we imagine that coagulated blood or tissue juices could only be removed with the greatest difficulty.

